# When Red Blood Cells Meet Carbon Monoxide: Yin and Yang in Medicines and Pharmaceuticals

**DOI:** 10.3390/ph19040634

**Published:** 2026-04-17

**Authors:** Taisei Nagasaki, Victor Tuan Giam Chuang, Masaki Otagiri, Kazuaki Taguchi

**Affiliations:** 1 Department of Pharmacy, Kumamoto University Hospital, Kumamoto 860-8556, Japan; t.nagasaki0507@gmail.com; 2 Pharmacy Discipline, School of Diagnostic and Therapeutic Sciences, Curtin University, Perth, WA 6845, Australia; v.chuang@curtin.edu.au; 3 Faculty of Pharmaceutical Sciences, Sojo University, Kumamoto 860-0082, Japan; otagirim@ph.sojo-u.ac.jp; 4 Graduate School of Biomedical and Health Sciences, Hiroshima University, Hiroshima 734-8553, Japan

**Keywords:** carbon monoxide, red blood cells, hemoglobin, drug delivery, modality

## Abstract

Carbon monoxide (CO) is a poisonous gas because it disrupts functional oxygen transport of red blood cell (RBC) by binding heme of hemoglobin with high affinity. Contrarily, endogenous CO, which is constantly generated in the process of heme degradation by heme oxygenase, functions as a gaseous mediator necessary for maintaining physiological homeostasis. This toxicological (Yin) and physiological (Yang) duality presents a distinctive problem in medical and pharmaceutical applications, prompting the central question of this review: How can strict control over CO’s exposure dynamics, magnitude, kinetics, and tissue context be achieved to enable its safe therapeutic use? Here, we integrate the Yin and Yang of CO through an innovative exposure-engineering framework, leveraging the inherent RBC characteristics to offer a novel conceptualization for therapeutic development. We highlight the role of native RBCs as a biologically grounded platform that can convert hemoglobin binding—classically viewed as the basis of CO toxicity—into a measurable and controllable buffering mechanism. Then, reconciling the Yin and Yang of CO based on RBCs enables medical and pharmaceutical modulation that is attractive for clinical situations, therapeutics and diagnostics. Finally, we discuss key translational challenges—local concentration control, patient-specific risk stratification, manufacturability and critical quality attributes, and regulatory positioning—and outline how quantifiable exposure control can enable the safe clinical development of RBC-based CO therapy.

## 1. Introduction

Carbon monoxide (CO) is classically recognized as a toxic gas because it binds to heme proteins such as hemoglobin and cytochrome oxidase with high affinity [1,2]. Excessive CO exposure can lead to harmful effects on the whole body and, in severe cases, fatal outcomes, making CO poisoning a major public health concern worldwide [3]. These properties define the toxicological “Yin” of CO, which has long dominated its medical perception. Notwithstanding its toxicological identity, CO is endogenously produced during heme degradation catalyzed by heme oxygenase (HO-1 and HO-2), where it functions as a gaseous signaling mediator with anti-inflammatory, anti-apoptotic, and cytoprotective effects [4,5,6]. This physiological aspect constitutes the “Yang” of CO and highlights its role in maintaining cellular and tissue homeostasis in the body. Importantly, the coexistence of toxicological (Yin) and physiological (Yang) aspects is not merely a conceptual paradox but represents a central challenge for medicines and pharmaceutical sciences: the need to impose therapeutic controllability on an intrinsically hazardous molecule. Unlike conventional drugs, from small-molecule drugs to biological products, gaseous mediators such as CO challenge traditional medical and pharmaceutical paradigms because of their rapid diffusion, limited intrinsic target specificity (in case of CO, it targets ferrous iron of heme group in biological systems), and narrow safety margins [7,8].

**Figure 1:**
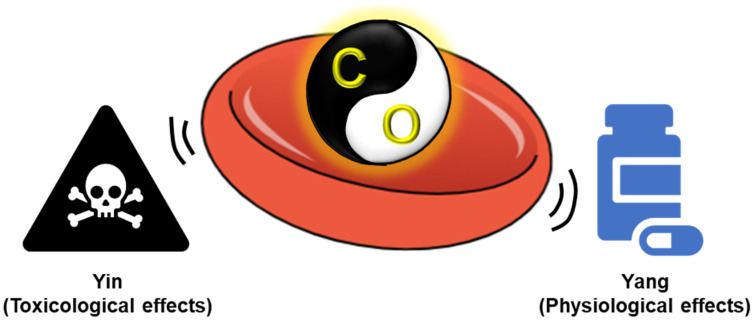
RBC-enabled exposure engineering reconciles the Yin–Yang duality of CO. The Yang aspect of CO at low concentrations supports its potential medical and pharmaceutical applications, whereas its Yin aspect at high concentrations limits its use due to toxicity in biological systems. RBC-associated properties of CO, such as buffering capacity, help reconcile these opposing effects, enabling the safe utilization of CO in medical and pharmaceutical contexts.

Red blood cells (RBCs) account for approximately 70% of all mammalian cells in the human body and, as highly mobile carriers, have access to virtually all tissues [9]. They have long been regarded as passive carriers of oxygen, dedicated primarily to gas transport between the lungs and peripheral tissues. This view has substantially expanded over the past few decades. RBCs are now recognized as dynamic regulators of vascular tone, redox balance, and intercellular signaling through interactions with nitric oxide, reactive species, and other mediators [10,11]. In addition, emerging evidence indicates that RBCs serve as key regulators of systemic glucose metabolism [12]. Through reversible binding CO to hemoglobin, RBCs possess an intrinsic capacity to buffer, transport, and modulate CO bioavailability [13,14]: CO bound to hemoglobin, which is referred to as carboxyhemoglobin (COHb), can dissociate and be delivered to tissues in a context-dependent manner, governed by the allosteric properties of hemoglobin. Hemoglobin exists in a high-affinity relaxed (R) state under oxygen-rich environments at high pH such as arteries and the lungs, and a low-affinity tense (T) state in oxygen-poor environments at a low pH such as peripheral tissues [15,16,17]. Importantly, hemoglobin’s affinity for CO decreases markedly from the R state (dissociation constants: 0.7–1.7 nM) to the T state (dissociation constants: ~1.1 μM), and this shift, together with modulators such as oxygen level, pH, and 2,3-diphosphoglycerate (2,3-DPG), promotes CO dissociation in tissues [18,19]. Although hemoglobin exhibits a substantially higher affinity for CO than for oxygen (approximately 240-fold) [20], this relationship is dynamically influenced by local physiological conditions, enabling the release and delivery of CO to target tissues. Structurally, RBCs are anucleate cells that lack organelles and exhibit a biconcave discoid shape that confers high deformability and prolonged circulation [21]. Moreover, advances in formulation science and bioengineering have enabled the rational design of RBC-based delivery systems with tunable circulation times, tissue distribution, and release kinetics [22,23]. These characteristics fundamentally shift the perception of RBCs from mere biological bystanders to engineerable carriers, uniquely capable of reconciling the Yin and Yang of CO within a medical and pharmaceutical framework, a perspective not fully explored previously (Figure 1).

This review provides medical and pharmaceutical perspective on the dual role of CO in RBC biology. Beyond simply cataloging biological effects, we focus on how pharmaceutical design principles—dose control, spatial regulation, and safety governance—can transform CO from a toxic gas into a therapeutically tractable modality. By conceptualizing RBC–CO interactions through the perspective of Yin and Yang, we aim to clarify the key challenges and opportunities in CO-based drug development and to highlight future directions for safe and effective medical and pharmaceutical applications.

## 2. Yin: Toxicological Aspects of CO (Figure 2)

From an RBC-centered perspective, CO exposure can perturb rheology and microvascular oxygen delivery by altering hemoglobin–oxygen kinetics, and through potential interaction with nitric oxide bioactivity and oxidative stress. Consequently, CO-induced dysfunction should be understood as a systemic disruption involving oxygen transport, mitochondrial bioenergetics, and vascular regulation.

**Figure 2:**
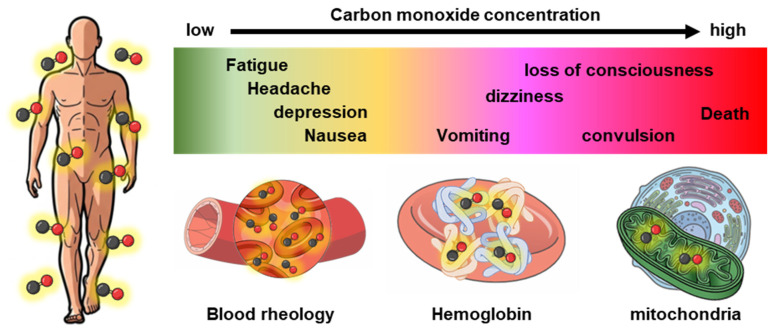
The Yin (toxicological) aspects of CO in biology. CO impairs blood rheology, hemoglobin function within red blood cells, and mitochondrial function in tissues, thereby inducing a range of clinical symptoms in a concentration-dependent manner, with severity increasing at higher concentrations.

### 2.1. Impaired RBC Rheology

CO poisoning has traditionally been framed as a consequence of COHb-mediated impairment of oxygen transport. The impact of COHb formation on RBC hemorheology, including viscosity and deformation, remains unclear. However, recent preclinical studies suggest that CO may affect viscosity, RBC deformability, and the fibrinolytic-coagulation system [24,25,26]. In ischemia-reperfusion model rats, CO exposure can also disturb RBC rheology and coagulation, contributing to acute toxicity and delayed neuropsychiatric effects [24]. In rabbit model of severe CO exposure with high COHb, RBC count, hemoglobin, and hematocrit increased, and whole-blood viscosity varied over time. Hemorheological indices indicated transient reductions in RBC deformability and aggregation, with values largely returning toward baseline within several days. In contrast, plasma viscosity and fibrinogen increased after exposure and remained elevated. These patterns are consistent with a superimposed acute-phase response and activation of coagulation-related pathways [25]. In a Wistar rat model, RBC deformability increased after CO exposure, and post-exposure oxygen regimens were reported to differentially affect rheological readouts [26]. These observations suggest that the direction and magnitude of CO-related hemorheological changes depend on species, exposure intensity, and assessment methodology, indicating a complex, non-uniform phenotype. While animal studies underscore this complexity, direct evidence in humans remains limited, highlighting a critical gap for future investigations. Furthermore, the net functional consequence of these hemorheological changes on oxygen delivery remains incompletely resolved. Available data suggest that reductions in RBC deformability and rises in whole-blood viscosity observed at elevated COHb levels may impair microvascular perfusion and oxygen offloading; however, the reported direction of these changes is not uniform across studies, and the threshold COHb levels at which clinically meaningful impairment occurs have not been established. These unresolved questions represent critical gaps for the safe clinical translation of RBC-based CO delivery systems and should be a focus of future mechanistic and translational investigations.

### 2.2. Impaired Oxygen Delivery of RBC

The toxicity of CO is most prominently manifested through its interaction with hemoglobin in RBCs, the most abundant heme proteins in blood. CO binds to hemoglobin with an affinity far greater than that of oxygen, forming COHb and competitively inhibiting oxygen binding [7]. Even modest increases in the COHb ratio in RBCs reduce the oxygen-carrying capacity of RBC, as reflected by a reduction in P50 (the oxygen tension which hemoglobin is 50% saturate), by stabilizing hemoglobin in a high-affinity (R) state, resulting in impaired oxygen release in peripheral tissues [27]. CO toxicity is not limited to hemoglobin-mediated hypoxia. CO can diffuse into cells and inhibit mitochondrial respiration, particularly at cytochrome *c* oxidase (complex IV), contributing to impaired oxidative phosphorylation, reactive oxygen species (ROS) generation and inflammatory signaling [28]. These combined mechanisms help explain why clinical severity does not always correlate tightly with COHb ratio and why delayed neurological sequelae can occur even after apparent clinical recovery [29,30]. Moreover, CO diffusion in peripheral tissues can affect the functions of other heme proteins, such as cytochrome P450 and NADPH oxidase, which partially contribute to maintaining homeostasis in the microenvironment [31,32]. Since the relationship between CO and mitochondria/cytochrome P450/NADPH is outside the scope of this review, detailed descriptions can be found in relevant reviews [18,31,33,34,35].

### 2.3. Carbon Monoxide Poisoning

Clinically, CO poisoning presents with a broad spectrum of symptoms that mainly reflect systemic hypoxia and cellular energy failure via the formation of COHb in RBC and the inhibition of mitochondrial respiration [36]. The symptoms are non-specific and worsen as the exposure duration and concentration increase. Early manifestations often include headache, dizziness, nausea, and confusion; severe cases can involve syncope, seizures, coma, and cardiovascular complications such as myocardial ischemia and arrhythmias [37]. The COHb level in the blood is a useful biomarker of CO exposure but is an imperfect surrogate for toxicity because it depends on exposure duration, time-to-sampling, supplemental oxygen before measurement, and host susceptibility (e.g., cardiopulmonary reserve, anemia, and pregnancy) [38]. CO-oximeters have been widely used for the assessment of CO poisoning worldwide through non-invasive measurement of the COHb derived wavelengths at the fingertip. However, recent meta-analysis concluded that CO-oximeters may be inadequate for estimating COHb in case of CO poisoning; therefore, CO-oximetry is required for accurate COHb quantification [39].

Treatment focuses on the rapid elimination of CO and the prevention of secondary injury. High-flow 100% oxygen accelerates the COHb clearance and is the first-line therapy [8]. Hyperbaric oxygen is considered for selected patients (e.g., severe neurological manifestations, elevated COHb level accompanied with high-risk clinical features) and is found to reduce the risk of neurologic sequelae [40]. However, the evidence and selection criteria for this therapy remain debated. A low-molecular synthesis antidote for CO poisoning has been investigated as an alternative to hyperbaric oxygen therapy [41].

## 3. Yang: Physiological Roles of CO (Figure 3)

### 3.1. Endogenous CO Production and Disposition

In contrast to its toxicological profile, CO is constitutively produced in mammalian tissues as a byproduct of heme degradation [5]. This endogenous production occurs at low and tightly regulated levels. In fact, the endogenous production rate of CO exceeds 16.4 μmol/h, resulting in a total daily output of approximately 12 mL of CO [42,43]. The degradation of senescent RBC is one of the major sources of endogenous CO production, as RBCs contain an abundance of hemoglobin, a hemeprotein. Furthermore, RBC are deeply involved in the disposition of endogenous CO: approximately 90% of the body’s total CO is bound to hemoglobin in circulating RBCs, and delivered to tissues as required [44]. The CO released from RBC functions as a gaseous transmitter and is a crucial substance for maintaining homeostasis via various physiological signaling pathways as described below.

**Figure 3:**
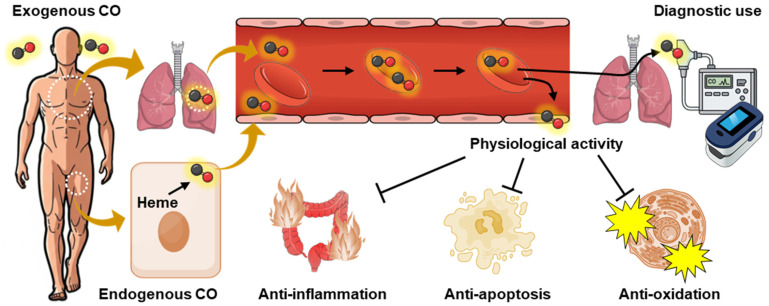
The Yang (physiological) aspects of CO and its applications. Low concentrations of exogenous CO absorbed through the lungs, as well as small amounts of endogenous CO generated during heme metabolism by heme oxygenase, bind to hemoglobin in red blood cells and circulate throughout the body. These CO molecules are subsequently released to exert physiological effects, including antioxidant, anti-inflammatory, and anti-apoptotic actions, thereby contributing to the maintenance of homeostasis. In addition, CO is utilized in pulmonary function testing and in the diagnosis of CO poisoning.

### 3.2. Physiological Signaling

Endogenously generated CO functions as a gaseous signaling mediator with broad cytoprotective potential [45]. In diverse experiments, low concentrations of CO have been associated with anti-inflammatory, anti-apoptotic, anti-proliferative, and antioxidant effects. (The detailed beneficial physiological effects of CO as a gasotransmitter can be found in relevant reviews [46,47,48].) These effects are complexly and complementary mediated via a wide variety of physiological signaling pathways, including modulation of MAPK cascades, partial engagement of soluble guanylate cyclase (sGC)–cGMP signaling, and suppression of pro-inflammatory transcriptional programs, and mitochondrial redox regulation.

MAPK signaling, particularly p38 MAPK and ERK1/2, is frequently implicated in CO-mediated protection. In inflammatory settings, CO attenuates cytokine production and promotes anti-inflammatory phenotypes, in part via IL-10 [4]. In the vasculature, CO can activate sGC/cGMP and modulate large-conductance Ca^2+^-activated K^+^ channels [49,50]. These effects support vasorelaxation and microcirculatory regulation, especially when nitric oxide bioavailability is impaired. Mitochondria represent another target of CO. CO can bind transiently to cytochrome *c* oxidase (complex IV). This interaction may induce mild respiratory modulation and controlled mitochondrial ROS (mtROS) signaling. mtROS signaling can activate adaptive programs such as NRF2-regulated antioxidant responses and HO-1 induction [51,52]. These observations are consistent with a mitohormetic model. CO also dampens apoptosis through multiple routes, including caspase inhibition, Bcl-2 family modulation, and reduced mitochondrial outer membrane permeabilization [6]. In addition, CO can suppress NF-κB and inflammasome-associated signaling [53].

The physiological significance of CO-mediated signaling has been demonstrated across a broad range of experimental disease models. In sepsis and endotoxemia models, such as lipopolysaccharide-induced systemic inflammation, exogenous CO administration attenuates pro-inflammatory cytokine release and improves survival outcomes [4]. In ischemia–reperfusion injury models involving the heart, liver, kidney, and lung, CO reduces tissue necrosis, preserves microvascular perfusion, and limits inflammatory cell infiltration [54]. Similarly, in transplantation models, CO has been shown to mitigate graft injury and improve functional recovery, supporting its potential relevance to ischemic and inflammatory graft pathology [55,56]. Moreover, in models of pulmonary hypertension and vascular remodeling, CO suppresses pathological smooth muscle proliferation and decreases vascular resistance, indicating a regulatory role in chronic vascular dysfunction [57].

### 3.3. Attractiveness of CO for Medical and Pharmaceutical Applications

The recognition of CO as an endogenous signaling mediator has stimulated extensive research on its therapeutic potential. However, the direct CO administration poses substantial medical and pharmaceutical challenges. The most investigated method for exogenous CO supply is the use of CO-releasing molecules (CORMs) and related CO-donor chemistries. In 2002, two types of CORMs were reported: CORM-1 and CORM-2 [58]. Subsequently, CORM-3, a derivative of CORM-2, was synthesized [59]. The development of these CORMs has led to dramatic advances in research on the physiological signaling of CO and laid the foundation for its medical and pharmaceutical applications. For example, CO supply from CORMs has shown in vivo efficacy in models of myocardial, hepatic and renal ischemia–reperfusion injury [59,60,61,62,63]. However, these early CORMs had low water solubility, short half-lives, and lacked target specificity, making them unsuitable for medical use. Therefore, various CORMs have been developed to provide localized or triggered CO delivery for disease treatment [64,65,66,67,68]. Although these approaches represent significant advances, translation has been constrained by challenges, including release kinetics control, off-target effects of carrier moieties (especially for metal carbonyls), and uncertainties regarding long-term safety. Crucially, multiple recent experimental studies have demonstrated that some CORMs, previously considered paradigmatic CO-releasing molecules, may release CO with specific factors or conditions in vitro [69,70,71,72,73,74,75], suggesting that CORMs are controversial as a reliable tool for in vivo studies. An extensive and comprehensive discussion of this issue was detailed in a recent review [76]. This finding significantly challenges the established understanding of their mechanisms and casts doubt on the usefulness of certain CORM types, underscoring the urgent need for re-evaluation in the field. Overall, these findings underscore a gap between biological efficacy and pharmaceutical feasibility, emphasizing the need for CO delivery platforms that enable precise dosing, spatial control, and safety governance within clinically acceptable margins.

### 3.4. Attractiveness of CO for Diagnostic Applications

CO is gaining interest as a diagnostic and imaging target because endogenous CO production largely reflects heme oxygenase activity, particularly stress-inducible HO-1. Practically, CO can be assessed using (i) CO-responsive optical probes for spatiotemporal imaging in cells and tissues [77], (ii) heme-based recognition sensors that exploit CO’s high affinity for ferrous heme [78], and (iii) exhaled (alveolar) CO as a non-invasive proxy of systemic CO generation [79]. Nevertheless, quantitative interpretation in vivo remains challenging because CO rapidly diffuses and is strongly buffered by hemoproteins. Accordingly, CO readouts may lack disease specificity unless integrated with complementary biomarkers of redox status, inflammatory and oxygenation.

A well-established clinical application is breath CO–based estimation of RBC lifespan [80]. Because most endogenous CO arises from heme degradation during physiological RBC turnover, alveolar CO measurements after correction for hemoglobin mass and key physiological factors can be used to estimate average RBC survival time [81]. This CO breath test concept is grounded in isotope-based approaches that link heme catabolism to CO generation [82] and has been applied to disorders with altered erythrocyte turnover, including newly diagnosed multiple myeloma [83], anemia [84], and clinical contexts relevant to HbA1c interpretation [85], providing a repeatable, nonradioactive tool for longitudinal monitoring. Another well-established diagnostic application is CO-oximetry, which non-invasively monitors COHb in blood to diagnose CO poisoning [86]. Multi-wavelength CO-oximetry enables quantification not only of COHb levels but also other types of hemoglobin (oxyhemoglobin, deoxyhemoglobin, and methemoglobin), making it possible to diagnose hypoxia and methemoglobinemia [87,88]. Importantly, CO-based diagnostics include both breath and blood measurements, but they are analytically distinct. Blood COHb is typically quantified by multi-wavelength spectrophotometry (CO-oximetry) that resolves the absorbance signatures of oxyhemoglobin, deoxyhemoglobin, COHb, and methemoglobin [89], whereas the breath CO test estimates RBC turnover by measuring endogenous CO in alveolar air under controlled sampling conditions. Accordingly, breath CO and blood COHb should not be used interchangeably. The former primarily reflects heme turnover/RBC lifespan. In contrast, the latter reflects recent systemic CO exposure and hemoglobin binding at the time of sampling.

## 4. RBC-Based CO Delivery: Reconciling Yin and Yang

**Figure 4:**
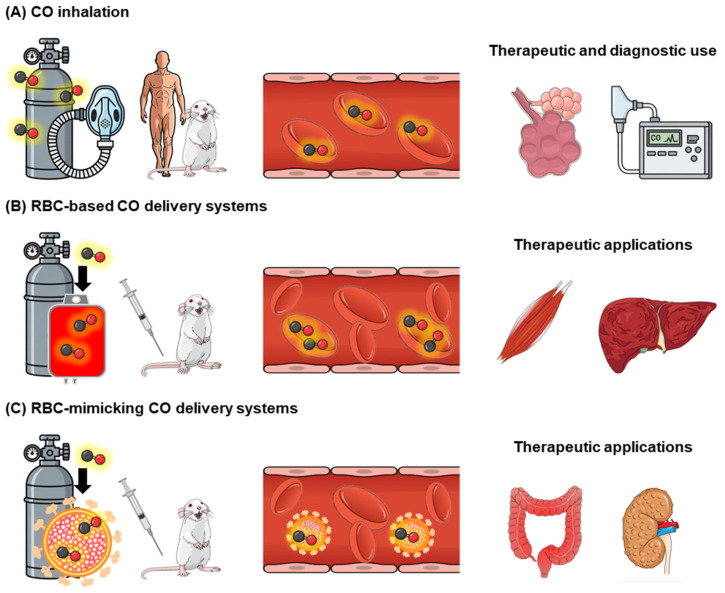
Schematic illustration of representative strategies for delivering CO to the body via RBCs. (**A**) Inhaled CO is absorbed through the lungs and binds to hemoglobin in RBCs, forming COHb that circulates systemically. While therapeutic effects have been demonstrated in various animal models, CO is clinically used in humans for pulmonary function testing and the diagnosis of CO poisoning. (**B**) Administration of CO-bound RBCs enables gradual CO release in peripheral tissues such as skeletal muscle and liver, where it exerts physiological effects, including antioxidant, anti-inflammatory, and anti-apoptotic actions, as demonstrated in various animal models. (**C**) CO delivery systems using RBC-mimicking carriers, such as hemoglobin vesicles, enable the delivery of CO to organs (e.g., intestine and kidney), where it exerts similar physiological effects in animal models.

The dual role of CO as both a toxic gas and a physiological signaling molecule is a practical constraint that demands medical and pharmaceutical control. Because COHb kinetics and mitochondrial effects can shift the balance from Yang to Yin with relatively small exposure changes, RBCs provide a biologically grounded framework for regulation by buffering CO through reversible hemoglobin binding. The representative examples for this are the utilization of endogenous RBC with CO inhalation, and the exogenous administration of RBC or RBC-mimicking nanoparticles that carry CO-bound hemoglobin, as describe below (Figure 4).

### 4.1. Inhalation CO

Inhaled CO can systemically deliver exogenous CO by binding to hemoglobin in RBC (Figure 4A); however, it lacks tissue specificity and carries an inherent risk of overdose, making precise dose control difficult in clinical settings. Studies in healthy volunteers in the 1970s reported no harmful effects after inhalation of 100 ppm CO for 8 h [90,91]. Considering these human results and the accumulated animal experimental data, CO inhalation at concentrations up to 500 ppm has been tolerated in mammals [92]. Early clinical studies have explored low-dose inhaled CO in selected populations (e.g., chronic obstructive pulmonary disease, idiopathic pulmonary fibrosis, and sepsis-induced acute respiratory distress syndrome) [93,94,95], illustrating both feasibility and the continued need for rigorous risk control. However, CO inhalation therapy has not reproduced in patients the remarkable therapeutic effects observed in animal models. Accordingly, the translational potential of CO inhalation therapy remains debatable.

Because inhaled CO exposure is determined by alveolar uptake and hemoglobin binding, pulmonary gas-transfer capacity is a key determinant of dose–exposure relationships. The diffusing capacity for CO (DLCO), which is routinely assessed for pulmonary function test, can be leveraged as a clinically available covariate to interpret inter-individual variability in CO uptake and COHb kinetics, particularly in early-phase studies [7,96]. Importantly, DLCO should not be treated as a surrogate for therapeutic exposure or safety. Reduced DLCO often reflects underlying parenchymal or vascular disease and limited oxygen reserve, which may increase vulnerability to hypoxemia even at comparable COHb levels. Accordingly, DLCO is positioned to complement direct exposure monitoring and rigorous cardiopulmonary/neurological safety surveillance within a monitoring-enabled dose-adjustment framework.

### 4.2. Administration of RBC-Bound CO

Against this backdrop, RBCs have emerged as a biologically informed and medically/pharmaceutically attractive platform for CO delivery. As endogenous CO carriers via hemoglobin binding, RBCs possess intrinsic mechanisms for buffering and transporting CO while minimizing abrupt systemic exposure. Advances in formulation and RBC engineering have expanded this concept into RBC-based and RBC-mimicking CO delivery systems (Figure 4B,C). As mentioned above, intact autologous RBCs themselves can serve as a physiological reservoir and carrier of endogenous CO. In the RBC-based CO delivery system the inherent properties of RBCs against the exogenous CO supply are utilized: CO is loaded onto hemoglobin within native RBCs in vitro, exploiting the intrinsically high affinity of hemoglobin for CO, along with the biocompatibility and circulation stability of RBCs [97]. Importantly, CO-RBCs reframe CO therapy as an exposure-control problem rather than a simple dose-delivery task, as therapeutic outcomes are governed by controllable parameters such as CO loading conditions, administered RBC mass, and in vivo CO exchange kinetics. As summarized in Table 1, preclinical studies using rodents have demonstrated that RBC-based CO delivery systems exert therapeutic and protective effects via physiological signaling actions in several disease-relevant models. The advantage is that systemic exposure can be quantitatively tracked through COHb time courses. In this context, COHb serves not only as a toxicity marker but also as a dynamic readout linking administered CO-bound native RBCs to pharmacokinetic exposure, oxygen delivery capacity, and downstream pharmacodynamic responses. This dual role of COHb highlights a key advantage of RBC-based CO delivery systems: the same hemoglobin-mediated mechanism that underlies CO toxicity (Yin) can be repurposed as a measurable and controllable mediator of therapeutic action (Yang).

Strategies for RBC-mimicking CO delivery systems include the use of hemoglobin vesicles, which are biomimetic preparations for RBCs and have been developed as RBCs alternatives [108]. CO-loaded hemoglobin vesicles have demonstrated proof-of-concept efficacy in several disease models, such as hemorrhagic shock [97], respiratory [109,110,111], renal [112], skin [113], and gastrointestinal disorders [114,115,116] (Table 2).

From a pharmaceutical standpoint, the value of RBC-based CO delivery lies not only in improved efficacy but also in the reconciliation of CO’s Yin and Yang by embedding CO within a biologically familiar carrier and applying design principles to control the dose, distribution, and release kinetics. Across delivery modalities, the biological outcome of CO is dictated not by its intrinsic signaling capacity but by how precisely systemic and local exposure can be regulated in space and time. Inhaled CO provides rapid systemic exposure with minimal spatial and kinetic control [117], whereas small-molecule CO donors can improve localization [118] but often suffer from environment-dependent release and off-target effects [76,119]. In contrast, RBC–based CO delivery embeds CO within a physiologically integrated buffering system via hemoglobin binding, enabling rational tuning of dose magnitude, release kinetics, and circulation time, which are key pharmaceutical control levers that define exposure profiles [97]. This exposure-centric framework transforms CO from a binary toxin–therapeutic dichotomy into a continuous spectrum, in which the therapeutic window can be actively engineered rather than passively tolerated. Furthermore, pill-based CO formulations, including CORMs and HBI-002, have been actively developed and have demonstrated potential for clinical application in animal models [120,121,122,123,124]. Following oral administration, blood COHb levels were increased, indicating that CO was absorbed and circulated systemically as COHb inside RBCs [125]. The oral route is the most commonly used and convenient method of administration, thus these formulations are expected to provide a feasible strategy for CO delivery. To date, some of these approaches have advanced to clinical trials (ClinicalTrials.gov: NCT03926819). A comparison of representative CO delivery strategies from an exposure engineering perspective is summarized in Table 3.

## 5. Challenge: Translating Yin and Yang into Medical and Pharmaceutical Design (Figure 5)

The effective translation of CO into therapeutics requires embedding toxicity management as a core design principle [126]. The biological activity of CO is inherently dose dependent. Thus, the central challenge is not whether CO is toxic or beneficial, but whether its exposure can be regulated in space, time, and magnitude with sufficient precision and safety margins. In addition, the clinical translation of CO will depend on whether pharmaceutical sciences can deliver predictable and governable exposure while meeting the operational demands of development and practice [127]. To more effectively repurpose RBCs as a CO delivery system in medicines and pharmaceuticals, progress will likely converge on six priorities: (i) formulation robustness and manufacturability, (ii) preservation and supplementation with formulation design, (iii) dosing strategy, (iv) safety and risk management, (v) translational strategy and regulatory alignment, and (vi) identification of clinical contexts in which CO’s pharmacology can offer meaningful benefits.

**Figure 5:**
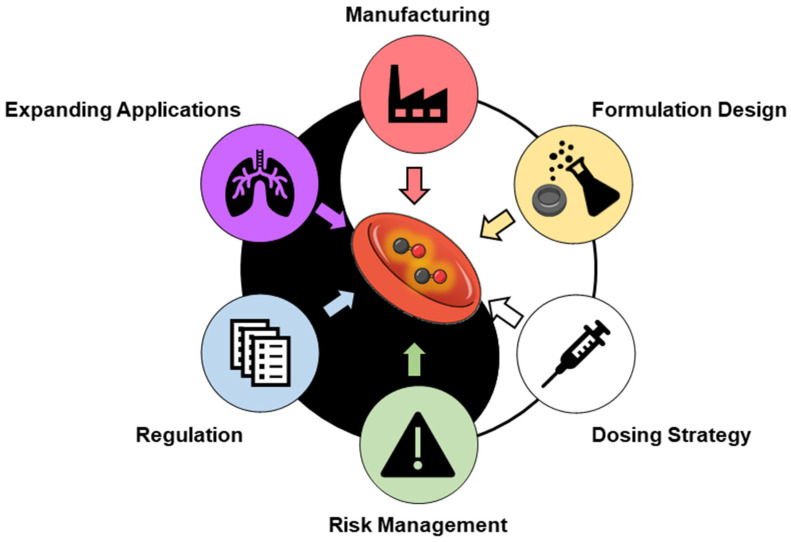
Priorities for RBC repurposing in CO applications in medicine and pharmaceuticals. This schematic highlights the key factors required for the successful repurposing of RBCs as CO delivery platforms. Critical priorities include ensuring safety through precise control of CO exposure, establishing robust manufacturing and quality control processes, optimizing formulation strategies, selecting appropriate routes of administration, and promoting clinical translation through regulatory alignment and evidence generation. Integration of these elements is essential for advancing RBC-based CO therapeutics.

### 5.1. Formulation and Manufacturing: Stability, Storage, and Quality Control

A formulation design that is stable, reproducible, and scalable, and whose critical attributes are measurable, is essential for medical and pharmaceutical applications. In advanced countries, RBC supply systems are already well established through organizations such as the Red Cross. CO-bound native RBCs can be readily prepared by exposing RBC concentrates to CO gas, without the need for specialized equipment. In addition, CO-bound native RBCs retained RBC quality for 42 days [128]. These facts suggest that the CO-bound native RBCs preparations can be stable and reproducible at scale. Furthermore, COHb was stable during storage [129], suggesting that COHb formation may prolong the storage duration without hemoglobin autooxidation. However, several key challenges still remain, including how CO-loading amount is retained during processing and storage, whether release kinetics remain stable over time, and how RBC integrity (membrane structure, deformability, and hemolysis risk) is preserved under clinically relevant handling conditions.

Quality control frameworks in the manufacturing process should evolve from single readouts to multi-parameter characterizations. Such characterization should integrate both biological and pharmaceutical attributes, including CO content and loading uniformity, CO release profiles, hemoglobin oxidation state, membrane integrity markers, and functional parameters relevant to the microcirculatory passage. The establishment of such quality control frameworks will be essential to simplify the preparation check and improve reproducibility between lots. Considering the characteristics of RBC and CO, establishing a simultaneous quality inspection system is extremely challenging.

### 5.2. Formulation Design

Spatial targeting introduces additional complexity because altered biodistribution could inadvertently concentrate CO in vulnerable tissues (e.g., myocardium or brain), where hypoxia sensitivity is high [130]. Thus, a key challenge in RBC-based CO delivery systems is precisely controlling the local CO concentration in target tissues. As native RBCs lack intrinsic mechanisms to direct CO release to specific sites, RBC-based formulation modification is a strategy to control CO release. Success will depend on whether the controlled CO release by buffering through reversible binding is preserved and supplemented with design features that ensure desirable behavior across clinically relevant conditions. Several RBC modification technologies have been developed and challenged for medical and pharmaceutical applications [131]. Membrane-modification strategies are optimized to avoid excessive accumulation in off-target tissues while maintaining sufficient exposure to elicit therapeutic signaling [132,133]. Furthermore, hypotonic dialysis, which enables the co-encapsulation of functional molecules into the RBC cytoplasm, is also a useful approach [134,135].

### 5.3. Dosing Strategies and Kinetic Control

Beyond formulation, dosing strategies represent a critical layer of control over CO delivery [126]. Optimal regimens should consider circulation time, CO release kinetics, and patient-specific factors, such as baseline hemoglobin levels, cardiopulmonary reserve, and comorbidities [7]. Rather than the conventional bolus paradigm, RBC-based CO therapies may require sustained or fractionated administration to maintain therapeutic signaling within safe margins. Kinetic control, which involves the regulation of CO association and dissociation from hemoglobin over time, should be considered a central design objective that links product attributes to clinical monitoring endpoints (e.g., COHb profiles and oxygen delivery surrogates) [136]. To date, optimal therapeutic CO concentrations have not been established for the prevention or treatment of any disorder.

Pharmacokinetic/pharmacodynamic (PK/PD) modeling is a commonly used approach to link blood concentration and therapeutic effects of various medicines [137,138,139]. However, comprehensive PK/PD modeling of CO has been challenging for existing CO donors, largely due to the elusive nature of CO’s precise PK/PD parameters. In terms of RBC-based or RBC-mimicking CO delivery systems, physiologically based pharmacokinetic (PBPK) modeling is required because the release of CO from hemoglobin is strongly influenced by the surrounding environment, such as pH and gas partial pressure [140,141]. Accordingly, it is desirable to develop formulations that incorporate quantitative PBPK/PD modeling and exposure–response analysis, ideally in a model-informed development framework.

### 5.4. Safety and Toxicity Risk Management: From “Acceptable Exposure” to “Quantifiable Control”

The narrow therapeutic window of CO requires that safety be defined in terms of quantifiable exposure thresholds rather than qualitative judgments. In clinical pharmacological terms, the therapeutic index of CO is unusually compressed: COHb levels considered tolerable in controlled settings (typically <~10%) lie in close proximity to levels associated with toxicity (≥15–20%) and severe poisoning (>25%) [94,142,143]. This proximity means that interindividual variability in CO metabolism—driven by differences in lung ventilation, hemoglobin mass, cardiac output, and endogenous CO production via heme oxygenase activity—can readily shift a patient from the therapeutic to the toxic range. However, safety must be engineered as a quantifiable exposure control, not asserted as acceptable exposure. Given CO’s narrow therapeutic margin, future development should consider exposure–response relationships that connect the administered dose, COHb kinetics, and tissue-level pharmacodynamics to both therapeutic endpoints and toxicity signals. For RBC-based CO delivery systems, safety evaluations should extend beyond systemic COHb levels. In addition, COHb consists of a diverse array of hemoglobin molecules each containing one to four CO molecules bonded (Hb_4_(CO)_4_, Hb_4_(O_2_)(CO)_3_, Hb_4_(O_2_)_2_(CO)_2_, Hb_4_(O_2_)_3_(CO), and Hb_4_(O_2_)_4_). This means that the ratio of the four Hb_4_(O_2_)*_n_*(CO)_4−*n*_ differs despite the blood level of COHb being identical. While the precise function of hemoglobin with varying numbers of bound CO molecules (Hb_4_(O_2_)*_n_*(CO)_4−*n*_) remains unclear, existing information indicates varying perspectives that have been addressed in other sources [144]. Elucidating these differences would enhance the safety and toxicity risk management of innovative RBC-based CO therapies and diagnostic approaches. Furthermore, it should also include indices of oxygen delivery and microcirculatory functions. This is important because toxicity may reflect the combined effects of hemoglobin-mediated hypoxia and tissue-level respiratory inhibition [37,145]. Model-informed approaches, patient stratification, and monitoring that support dose adjustment can help to operationalize safety [146].

### 5.5. Translational and Regulatory Considerations: Positioning RBC-Based CO as a Therapeutic Modality

CO-based therapies face a unique translational hurdle. CO is neither a conventional small molecule nor a traditional biologic, and RBC-based delivery further blurs the categorical boundaries. Early alignment with regulatory expectations regarding product classification, manufacturing controls, and risk mitigation plans is critical. In addition, translation of Yin and Yang of CO highlights a broader role for medical and pharmaceutical sciences: rendering inherently hazardous agents usable through control, predictability, and accountability. For RBC-based CO delivery systems, this entails quality-by-design (QbD) thinking to define critical quality attributes, critical process parameters, and risk mitigation plans. Practically, this also requires a monitoring strategy that links product attributes to clinically interpretable exposure and safety endpoints, and a development plan aligned with regulatory expectations for complex modalities, which may not fit neatly into traditional small-molecule or biologic categories. Collectively, these considerations indicate that the feasibility of CO therapeutics hinges on a pre-specified control strategy—spanning product design, dosing governance, and monitoring—rather than on biological potency alone.

Early phase trials may benefit from adaptive or model-informed dosing designs guided by COHb kinetics and pharmacodynamic biomarkers, coupled with rigorous cardiopulmonary and neurological safety monitoring. For clinical adoption, it will be essential to demonstrate clear differentiating advantages, such as improved controllability relative to inhaled CO or reduced off-target effects compared with CORMs and other CO donors. In parallel, the identification of biomarkers that reliably reflect CO toxicity, together with the development of clinically feasible measurement methods, remains an important unmet need.

### 5.6. Expanding Pharmaceutical Applications: Where CO Biology Meets Clinical Need

The most promising clinical applications of CO are likely those in which its pharmacology is mechanistically aligned with disease biology and where exposure can be reliably monitored. Diseases driven by inflammation and oxidative stress, such as ischemia–reperfusion injury and organ protection in transplantation settings, remain compelling targets [147,148]. RBC-based platforms offer a route to exposure-shaped CO signaling, enabling localized or sustained, low-level delivery while maintaining safety margins. This exposure control creates room for rational combination therapies (e.g., co-delivery with anti-inflammatory or antioxidant agents) and engineered targeting strategies that concentrate the benefits where CO biology is needed most. The key requirement remains accountability: biodistribution and tissue exposure must be quantifiable, and controllable, to a degree sufficient for dose adjustment and safety governance.

## 6. Future Perspectives and Conclusions

CO exhibits a unique duality as both a toxic gas and a physiological signaling molecule, presenting significant challenges and opportunities for medical and pharmaceutical applications. The future of CO-based therapeutics hinges on the precise control of its exposure, balancing its toxicological “Yin” with its physiological effects “Yang.” RBCs, with their intrinsic ability to reversibly bind and buffer CO via hemoglobin, offer a biologically grounded and engineerable platform to achieve this balance. By leveraging RBCs’ natural properties alongside advanced formulation and delivery technologies, it becomes possible to design CO therapies that maximize therapeutic effects while minimizing toxicity through controlled dose, spatial distribution, and release kinetics. Advancements in RBC-based CO delivery systems show promise in addressing the limitations of inhaled CO and CORMs by providing tunable pharmacokinetics and enhanced safety profiles. However, clinical translation requires overcoming challenges related to formulation stability, manufacturing reproducibility, preparation quality control, precise dosing strategies, and comprehensive safety management. Accompanied with these, it is urgent to ensure that the disorder-dependent threshold and biomarkers reflect the toxicity, and to establish clinically feasible monitoring techniques for toxicity. Beyond therapeutic applications, RBC-based CO platforms have the potential to revolutionize related fields, including antidote development for CO poisoning, diagnostic innovations, and fundamental research into CO’s biological roles. This multifaceted approach could establish CO not only as a novel treatment modality for difficult-to-treat diseases but also as a versatile tool across medical and life sciences.

In summary, the successful clinical adoption of CO therapies depends on the establishment of precise, quantifiable exposure control strategies rooted in RBC biology and medical/pharmaceutical design principles. Continued research into the complex interactions between RBCs and CO will further elucidate the mechanisms needed to harness CO’s full therapeutic potential safely. This progress is expected to transform CO from a traditionally feared toxin into a sophisticated, controllable therapeutic agent with broad clinical impact.

## Figures and Tables

**Table 1:** Representative preclinical disease models evaluated with RBC-based CO delivery systems.

Disease Model	Key Outcomes (Efficacy)	Mechanism	Refs.
Hemorrhagic shock	Reduced plasma markers of hepatic and renal injury Preserved hemodynamics and improved short-term survival	Reduced organ oxidative damage	[97,98]
Hepatic ischemia–reperfusion injury after massive hemorrhage and resuscitation	Preserved hepatic cytochrome P450 isoforms (1A2/2C11/2E1/3A2) expression and activity Attenuation of hepatic injury after resuscitation	Reduced free heme–associated hepatic oxidative injury Inhibited Kupffer cell activation Reduced IL-6/TNF-α production via HMGB1/TLR4 suppression	[99,100,101]
Rhabdomyolysis/crush syndrome–related acute kidney injury	Suppressed acute kidney pathogenesis and improved mortality Attenuated muscle injury and kidney injury	Suppressed heme-protein–associated renal oxidative injury such as myoglobin oxidation, free heme-linked oxidative injury Reduced systemic inflammation	[102,103]
Renal ischemia–reperfusion acute kidney injury	Suppressed pathogenesis of kidney injury Prevented progression to renal fibrosis/epithelial–mesenchymal transition	Upregulated HIF-1α, AMPK phosphorylation, and Nrf2 activation Promoted mitochondrial ROS signaling	[104]
Cisplatin-induced acute kidney injury	Attenuated kidney dysfunction and tubular injury	Reduced oxidative stress markers Reduced TNF-α/IL-6 and immune-cell infiltration	[105]
Metabolic dysfunction-associated steatohepatitis	Ameliorated MASH progression	Improved hepatic inflammatory and metabolic abnormalities Enhanced AMPK activity Inhibited Kupffer cell activation	[106]
Sarcopenia	Improved skeletal muscle dysfunction/sarcopenic phenotype	Exercise mimetic–like effects Improved redox/inflammatory muscle microenvironment and mitochondrial adaptive signaling	[107]

**Table 2:** Representative preclinical disease models evaluated with RBC-mimicking (hemoglobin vesicle) CO delivery systems.

Disease Model	Key Outcomes (Efficacy)	Mechanism	Refs.
Hemorrhagic shock	Reduced plasma markers of organ injury	Reduced organ oxidative damage	[97]
Obliterative bronchiolitis	Ameliorated allograft luminal occlusion and fibrosis	Suppressed M1 macrophage activation in tracheal allografts Decreased IL-17A production. Decreased the expression of TNF-α and TGF-β	[109]
Bleomycin-induced pulmonary fibrosis	Suppressed the progression of pulmonary fibril formation Improved respiratory function	Decreased ROS generation by inflammatory cells, NADPH oxidase 4 Decreased the production of inflammatory cells, cytokines and transforming growth factor-β	[110]
Acute lung injury	Suppressed pathogenesis of acute lung injury	Decreased ROS generation vis NADPH oxidase 4 Decreased the activation of the TLR4/NF-κB signaling pathway Suppressed the macrophage polarization toward M1-like macrophages	[111]
Cisplatin-induced acute kidney injury	Attenuated nephrotoxicity without failure of cancer treatment	Reduced apoptosis via caspase-3	[112]
Thermal skin damages by dye laser irradiation	Reduced burn injury after pulsed-dye laser irradiation	Reduced inflammation Reduced oxidative damage	[113]
Acute pancreatitis	Suppressed pathogenesis of pancreatic injury Prevented progression of distal organ injury (liver, kidneys, and lungs)	Inhibiting the production of systemic proinflammatory cytokines Suppressed the neutrophil infiltration, and oxidative injuries in pancreas Polarized macrophages toward an M2-like phenotype	[114,115]
Colitis	Improved colitis symptoms (weight loss, stool consistency, occult blood) Attenuated colonic histological damages Prolonged survival time	Suppressed a neutrophil infiltration Decreased the production of pro-inflammatory cytokines Attenuated oxidative injuries	[116]

**Table 3:** Comparison of CO delivery strategies.

Delivery Modality	Exposure Controllability	Key Strengths	Principal Limitations	Translational Considerations
Inhaled CO	Low (systemic, rapid)	Simple administration	Narrow safety margin	Restricted to controlled clinical settings
Pilled CO	Moderate (Predictable systemic CO exposure)	Simple administration (oral administration)	Variable kinetics (Lack of organ-specific delivery)	Regulatory complexity
CO-releasing molecules	Moderate (chemistry-dependent)	Potential localization	Variable kinetics, carrier effects	Regulatory complexity
CO-bound native RBCs	High (buffered)	Physiological integration	Cell handling and storage	Alignment with transfusion practice
hemoglobin vesicles (RBC mimicking)	High (tunable)	Scalable design	Artificial carrier complexity	Manufacturing reproducibility

## Data Availability

No new data were created or analyzed in this study. Data sharing is not applicable to this article.

## Abbreviations

The following abbreviations are used in this manuscript:

CO	Carbon monoxide
COHb	Carboxyhemoglobin
CORMs	CO-releasing molecules
DLCO	diffusing capacity for caron monoxide
mtROS	Mitochondrial reactive oxygen species
PBPK	physiologically based pharmacokinetic
PK/PD	Pharmacokinetic/pharmacodynamic
ROS	Reactive oxygen species
RBC	Red blood cell
sGC	soluble guanylate cyclase
